# Advantages and Possibility of Transcription Factors FOXOs in HCC

**DOI:** 10.1002/cbf.70126

**Published:** 2025-09-26

**Authors:** Gu Xiufeng, Hu Zhiping, Wang Jingzhi

**Affiliations:** ^1^ Hubei Provincial Hospital of Integrated Traditional Chinese and Western Medicine Wuhan Hubei China; ^2^ Department of Integrated Traditional Chinese and Western Medicine, Hubei Cancer Hospital, Tongji Medical College Huazhong University of Science and Technology Wuhan Hubei China; ^3^ Hubei University of Chinese Medicine Wuhan Hubei China

**Keywords:** FOXOs, HCC, tumor, tumor angiogenesis, tumor energy metabolism

## Abstract

The forkhead frame O protein (forkhead box class O proteins, FOXOs) is a highly conserved family of transcription factors, consisting of four members: FOXO 1, FOXO 3, FOXO 4, and FOXO 6. The FOXOs protein family is a ubiquitously expressed transcription factor involved in the regulation of cell cycle, cell proliferation, apoptosis, autophagy, DNA repair, antioxidative stress, and many other biological activities and plays a very important role in both physiological and pathological aspects. It is noteworthy that recent studies show that FOXOs have a significant role in the occurrence and development of tumors, particularly in primary hepatocellular carcinoma (hepatocellular carcinoma, HCC), it possesses numerous advantages. In this paper, we will summarize the structural and functional characteristics of FOXOs and summarize their tumor role and advantages in HCC, to provide new ideas for HCC research and promote the generation of more favorable prevention and treatment strategies.

## Summary

The study of related literature clearly demonstrates that FOXOs have unique potential in HCC. This expands the theoretical research related to FOXOs and HCC. It also explores the advantages and potential of FOXOs in HCC. This will provide new ideas and methods for academic research in this direction.

## 1 Overview

### 1.1 Epidemiology of Primary Hepatocellular Carcinoma

Liver cancer is a serious health problem threatening the world. According to the statistics of global cancer in 2020, liver cancer is the sixth malignant tumor in the world in terms of incidence rate and the third in terms of mortality, and the incidence and mortality rates are increasing year by year [1, 2]. In 2020, there were about 906,000 new cases and 830,000 deaths worldwide, and the estimated number of new cases is expected to exceed 1 million by 2025 [3, 4]. Liver cancer is one of the three causes of cancer deaths in 46 countries, and one of the five causes of cancer deaths in 90 countries. It is expected that the number of new cases of liver cancer will increase by 55.0% annually from 2020 to 2040, and by 2040, there may be 1.4 million people affected and 1.3 million people dying from liver cancer [5, 6]. Liver cancer has high malignancy, rapid progression, a low early diagnosis rate, and a low 5‐year survival rate. It brings great challenges to the medical and health system, seriously threatens people's lives and health, and also brings a heavy burden to the economy. Although the treatment of liver cancer involves surgery, TACE (Transcatheter Arterial Chemoembolization, TACE), radiotherapy, chemotherapy, targeted therapy, immunotherapy, etc., the overall survival period is only 23 months, and the 5‐year survival rate is only 11.7% to 14.1% [7]. FOXO is a highly conserved transcription factor that has unique potential in HCC according to the known action properties of FOXOs. In this review, we review the role of FOXO and its advantages in HCC in order to provide new therapeutic ideas.

### 1.2 Introduction to the FOXO Protein Family

The transcription factor forkhead frame O protein (Forkhead box class O proteins, FOXO) belongs to the O subgroup of the forkhead frame (Forkhead box, FOX) protein family. The FOX protein family consists of 19 subfamilies of transcription factors that share about 110 amino acid residues of a highly conserved DNA‐binding domain, also known as the winged coil domain. Although closely related to other subgroups, FOXO is the most divergent of the FOX family, because its forkhead domain within helix H3 is preceded by a unique five‐amino acid insertion, enabling direct sequence‐specific interaction with the DNA binding site. There are four members of the FOXO family: FOXO 1, FOXO 3, FOXO 4, and FOXO 6 [8, 9]. They have different expressions in different tissues. FOXO 1, FOXO 3, and FOXO 4 are widely expressed in all organs and tissues, and FOXO 6 is only expressed in the central nervous system due to its restrictive expression pattern [10]. FOXO proteins not only act as transcriptional activators but also as transcriptional inhibitors. Thus, this transcription factor family may have both positive and negative effects on gene expression, depending on the promoter environment and extracellular conditions. FOXOs play an important role in human physiological and pathological processes, regulating cell cycle, cell proliferation, apoptosis, autophagy, tumor suppression, energy metabolism, DNA repair, antioxidant stress, and more [9, 11, 12, 13].

## 2 The Structure, Function, and Role of FOXOs Proteins in Tumor Biology

FOXOs belong to the highly conserved transcription factor FOX protein family, characterized by the presence of a highly conserved wing‐shaped helix DNA binding domain (DNA Binding Domain, DBD) located in the N‐terminal region of DNA, as well as a nuclear localization signal (Nuclear Localization Signal, NLS), nuclear output signal (Nuclear Export Signal, NES), and transactivation domain (Transactivation Domain, TAD) [14, 15, 16]. These signal sequences enable FOXOs to shuttle between the nucleus and cytoplasm [17]. Among them, FOXO6 lacks NES, and its distribution within the nucleus does not depend on external signals regulated [18]. As FOXOs are important transcription factors shuttling between cytoplasm and nucleus, they can regulate cells through transcription‐dependent or transcript‐independent regulatory mechanisms and coordinate gene expression and activity through protein‐protein, protein‐DNA, and protein‐RNA interactions. The expression and activity regulation of FOXOs mainly include transcriptional and translational regulation [15, 17, 19, 20], posttranslational modifications (Protein translational modifications, PTMs) [21, 22], and protein‐protein interactions (Protein‐Protein Interactions, PPIs) [21, 23, 24, 25, 26, 27, 28, 29].

FOXOs play an important role in human physiological and pathological processes, regulating cell cycle, cell proliferation, apoptosis, autophagy, tumor suppression, energy metabolism, DNA repair, antioxidant stress, and more [9, 11, 12, 13]. Due to the indispensable importance of FOXOs in aspects such as cellular regulation, in recent years, their great potential in cancer prevention and treatment has been gradually explored. A large number of reliable studies have shown that FOXOs play a very important role in the occurrence, progress, metastasis, and recovery of hepatocellular carcinoma [30], soft tissue sarcoma [31], acute myeloid leukemia (Acute Myeloid Leukemia, AML) [32], prostate cancer [33], breast cancer [34, 35, 36], neuroblastoma [37], colorectal cancer [38], renal clear cell carcinoma [39], diffuse large B‐cell lymphoma [40], and other tumor diseases, but its specific mechanism has not yet been completely clear.

It is well known that cell proliferation begins in the quiescent state (also known as G0 phase), then from the G1 phase to the S phase, to the G2 phase, to the M phase. Numerous studies have shown that FOXOs can induce tumor cell cycle arrest while inhibiting tumor occurrence and development, and are important regulators of inhibiting cell proliferation [41]. Reliable studies have shown that activation of FOXO 1 induces apoptosis, cell proliferation arrest, and decreased cell viability in cervical cancer cell lines (HeLa, SiHa, ME‐180, and SW 756), while activation of FOXO 1 and its nuclear sequestration are crucial in the regulation of cervical cancer cell proliferation, cell viability, and apoptosis [42]. Meanwhile, FOXO 1 can induce hepatocarcinoma cell lines SMMC‐7721 and Bel‐7402 in the G0/G1 phase [43]. In addition, a large number of studies have shown that FOXOs can regulate the cell cycle [16] by regulating cyclin D1 and D2 [44, 45], Cyclin‐dependent kinases (CDKs) inhibitors, including p27kip1, p21Cip1, p15, p19, and p130 [46, 47], DNA damage‐induced protein 45 (DNA damage‐inducible protein 45, GADD45) [48], etc. Other studies have shown that inhibition of the PI3K (Phosphatidylinositol 3‐kinase, PI3K) /AKT (Protein Kinase B, AKT) signaling pathway or ectopic overexpression of FOXOs leads to cell cycle arrest of [49, 50] in cancer cell lines, including colon cancer, glioblastoma, and acute T cell leukemia.

One of the other important features of cancer cells is the ability to evade apoptosis, so studying the mechanism of induced apoptosis can provide possible therapeutic ideas for malignant tumors. While FOXOs can regulate the expression of several proapoptotic regulators, apoptosis is induced by multiple cellular endogenous and exogenous signals [16, 51]. Such as inducing endogenous cell apoptosis by regulating Bc1‐2 family members Bim (Bcl‐2 interacting mediator of cell death, Bim), Bcl XL (B‐cell lymphoma‐extra large, Bcl XL), BNIP3(BCL2 Interacting Protein 3, BNIP3), and Puma(p53 upregulated modulator of apoptosis protein, Puma) [52, 53, 54], and activating exogenous cell apoptosis by regulating the expression of fatty acid synthase ligand (fatty acid synthase‐ligand, Fasl), tumor necrosis factor related apoptosis inducing ligand (TNF related apoptosis inducing ligand, TRAIL), and TNF receptor associated death domain (TNFRSF1A associated via death domain, TRADD) [46, 55]. Additionally, studies have shown that the apoptosis pathway mediated by FOXOs is also associated with chemotherapy sensitivity [56, 57].

Autophagy is a self‐protective mechanism mediated by lysosomes occurring in cells, which involves intracellular degradation and removal of damaged proteins and organelles to maintain cell renewal and homeostasis, participating in the physiological and pathological processes of the body [58]. Many studies have found that the dysfunction of autophagy will lead to the generation of abnormal metabolites and the accumulation of normal metabolites, and then affect the occurrence of a variety of human diseases, including tumors [59, 60, 61, 62]. FOXOs, as transcription factors for nucleoplasmic shuttling, have been shown to regulate autophagy through both transcription‐dependent and transcription‐independent mechanisms. When located in the nucleus, FOXOs are involved in the transcription of several autophagy genes, and when translocated to the cytosol, FOXOs can also regulate autophagy [13, 14, 63] by directly interacting with cytosolic autophagy proteins. During muscle atrophy, FOXO3 upregulates several autophagy‐related genes, such as LC3 (Microtubule ‐ associated protein light chain 3, LC3), BNIP3, Gabarapl1 (GABA Type A Receptor Associated Protein Like 1, Gabarapl1), VPS34 (Vacuolar protein sorting 34, VPS34), ULK2 (Unc‐51 Like Autophagy Activating Kinase 2, ULK2), and ATG12 (Autophagy Related 12, ATG12) [14, 64, 65]. In addition, FOXO 3 can activate gene ATG 7 (Autophagy Related 7, ATG7) transcription and activate autophagy, further regulating the occurrence and development of lung cancer [66]. In addition, when cells face an energy crisis, dephosphorylated FOXO 3 enters the nucleus and activates SNAI 2 (Snail Family Transcriptional Repressor 2, SNAI2) transcription, which subsequently promotes the expression of autophagy genes PIK3CA (Phosphatidylinositol‐4,5‐Bisphosphate 3‐Kinase Catalytic Subunit Alpha, PIK3CA) and ULK1(Unc‐51 Like Autophagy Activating Kinase 1, ULK1) [67]; studies have also shown that FOXO 3‐induced autophagy in FOXO 1 is highly dependent on the transcriptionally active [68] FOXO 1. Other studies showed that FOXO can regulate autophagy [63] by a transcription‐independent mechanism. When cells are in an oxidative stress or starvation state, the acetylation modification of FOXO1 in the cytoplasm is enhanced. Subsequently, the acetylated FOXO1 specifically binds to the ATG7 protein, ultimately inducing autophagy. This autophagy mechanism is involved in regulating the occurrence and development of human colon cancer [69]. In addition, autophagy induced by FOXOs plays an important protective role in other tissue homeostasis, such as liver [70], brain [71, 72], and kidney [73], as well as disc and cartilage homeostasis [74, 75].

Oxidative stress is a state of excessive oxidation caused by the imbalance between oxidation and antioxidants in the body. It is mainly caused by the excessive production of reactive oxygen species (ROS) or the insufficient endogenous antioxidant capacity. Under normal circumstances, they are in a dynamic equilibrium state to protect the structure and function of cells. Reactive oxygen species (ROS) and cellular oxidative stress are involved in many physiological and pathological processes, including cell and body aging, death, inflammation, tumors and others [76, 77, 78]. The role of FOXOs against oxidative stress has been widely recognized [10, 39, 40]. For example, triple deletion of FOXO 1, FOXO 3, and FOXO 4 was found to accelerate tumor susceptibility and progression in mice, along with increased levels of intracellular ROS and abnormal regulation of cell cycle and apoptosis [79, 80], further suggesting that FOXOs play an indispensable role in maintaining cell homeostasis. In addition, FOXOs enhance the antioxidant capacity of cells [81, 82] by inducing genes that eliminate ROS and improve mitochondrial redox reduction by regulating manganese superoxide dismutase (MnSOD) [83], catalase [84], and GADD45 (Growth arrest and DNA damage‐inducible 45, GADD45) [85].

As is known to all, DNA is the most important genetic material of the human body; therefore, to ensure the integrity of DNA replication is crucial to life, many diseases, including the occurrence of tumors are closely related to the genome instability [86], but in the life cycle of organisms, a variety of exogenous and endogenous damage factors will make damage to DNA [87], so DNA damage repair mechanism is crucial to maintaining genome integrity and stability. Studies have shown that FOXOs can promote DNA damage repair. On the one hand, FOXOs can reduce DNA damage [81, 82] through the effect of resisting oxidative stress, and on the other hand, FOXOs can also bind to DNA to promote DNA damage repair [85, 88]. There are reliable studies showing that FOXO3a can regulate DNA double‐strand break repair and maintain genome stability while inhibiting mutagenesis and promoting the repair [89] of damaged DNA. In addition, FOXO3a can also promote homologous recombination repair (Homologous Recombination Repair, HRR) [90] by regulating the transcriptional activation of MRE 11 (MRE11 double strand break repair nuclease, MRE11), BRCA 1 (BRCA1 DNA Repair Associated, BRCA1), BR IP1 (BRCA1 Interacting DNA Helicase 1, BRIP1), and RAD 50 (RAD50 Double Strand Break Repair Protein, RAD50).

## 3 The Antitumor Role and Advantages of FOXOs in HCC

As mentioned above, FOXOs have a vital role in regulating cell cycle, cell proliferation, apoptosis, autophagy, DNA repair, antioxidative stress, and other [9, 11, 12, 13]. It is precisely because of the indispensable importance of FOXOs in cell regulation and other aspects that their huge potential in tumor prevention and treatment is being gradually explored and verified in recent years. Also, FOXOs in the physiology and pathology of the liver, and primary liver cancer is a common malignant tumor, including hepatocellular carcinoma (HCC), which accounts for 70–80% [91]. Combining the functional roles of FOXOs, in addition to the aforementioned effects, they also have multiple advantages in the prevention and treatment of hepatocellular carcinoma (HCC), such as regulating energy metabolism, immune environment, and angiogenesis, etc.

### 3.1 The FOXOs Are the Effector Molecules

**Figure 1:**
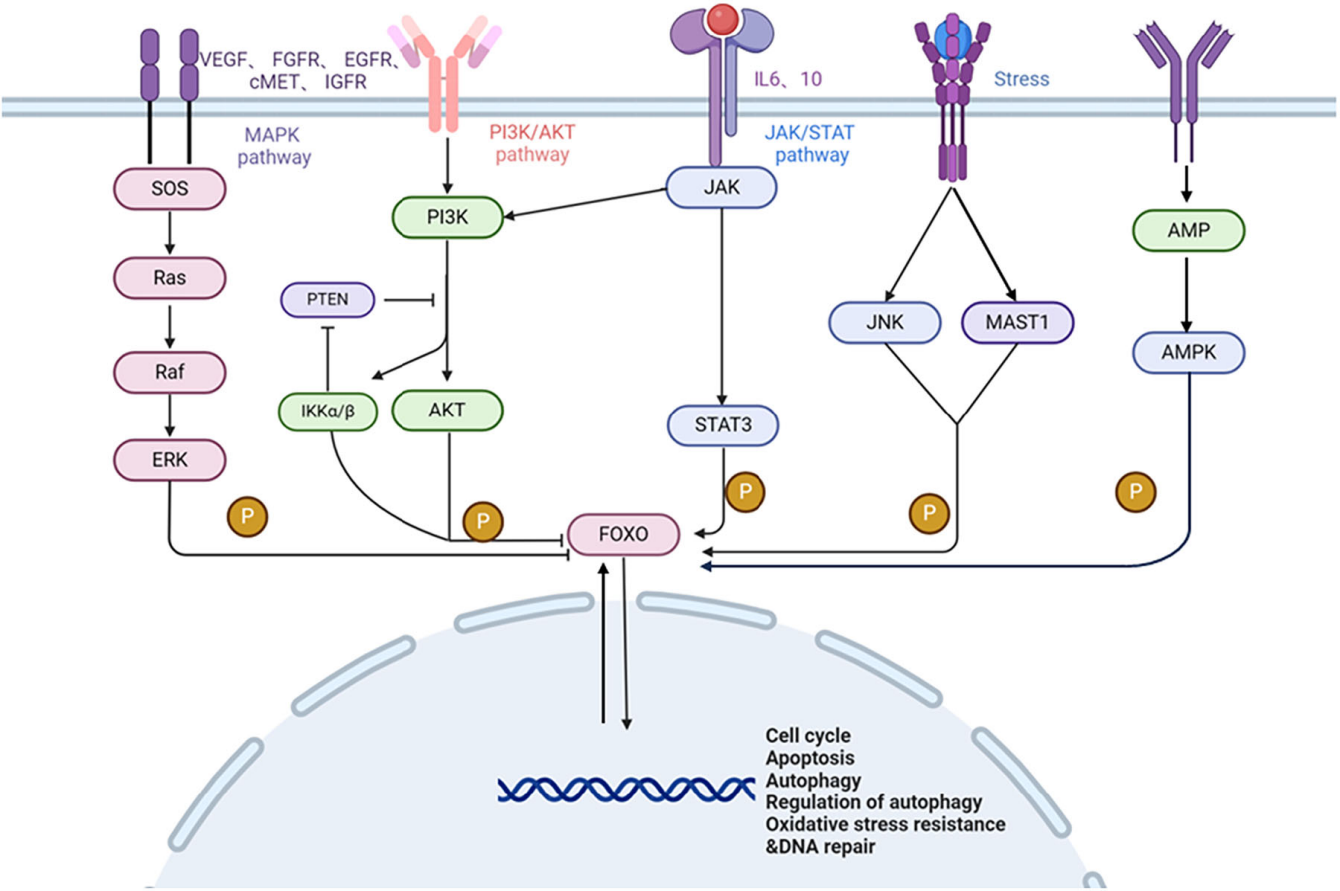
FOXOs‐related HCC signaling pathway.

As is known to all, in the case of changing external conditions, the successful adaptation and long‐term survival mainly depend on the precise regulation of gene expression and transcription factors, as the final effectors are recruited to specific regions of target genes to regulate [92]. FOXOs are highly conserved transcription factors, and they are also important regulatory links and effector molecules in the signaling pathways of multiple hepatocellular carcinomas, such as PI3K/AKT, Ras/Raf/MAPK (Rat Sarcoma virus/Rapidly Accelerated Fibrosarcoma/Mitogen‐Activated Protein Kinase, Ras/Raf/MAPK), JAK/STAT (Janus Kinase/Signal Transducer and Activator of Transcription, JAK/STAT) (Figure 1) [93] et al. Abnormal activation of signaling pathways such as PI3K/AKT phosphorylates FOXOs, causing them to migrate from the nucleus to the cytoplasm, thereby losing transcriptional activity and normal regulation of the cell.

### 3.2 Regulation of Tumor Energy Metabolism

The continuous proliferation of tumor cells cannot be separated from the support of energy, and it is precisely because of the unique characteristics of the energy metabolism of tumor cells that they can survive and proliferate in the harsh microenvironment. It is well known that the liver has a vital role in the body's energy metabolism, including glucose metabolism, protein metabolism, fat metabolism, etc., to maintain and regulate the lipid and glucose levels in the body. Recently, with the deepening study of FOXOs, it was revealed that FOXOs are crucial for the function of the liver and have important regulatory roles in key energy metabolism pathways such as glucose, amino acids, and lipids, including the regulation of tumor energy metabolism.

It has been shown that in normal physiology, FOXO 1 is the main target of insulin, and the transcriptional activity is inhibited by nuclear displacement; the liver is one of its key sites of action [94, 95]. FOXO 1 can promote hepatic glucose production and regulation of lipid metabolism, so under insulin resistance, it causes hyperglycemia and dyslipidemia [94]. When FOXO 1 is highly expressed in the liver, fasting blood glucose increases [96], and liver‐specific FOXO 1 knockout mice will have hypoglycemia [97]. When all three types of FOXOs are knocked out in the liver, glucose metabolism becomes more disordered than in single liver FOXO1 knockout mice [98]. The mechanism behind this is related to the regulation of FOXOs by AKT. Interestingly, in the absence of AKT, FOXO1 can still maintain blood glucose stability, while FOXO1 does not inhibit the upregulation of insulin‐mediated synthesis metabolism processes such as glycogen and lipid synthesis [99].

Glucose is also a major energy source for cancer cells and a key nutrient to support biosynthesis in tumors, but the increased ability of cancer cells to absorb glucose from the extracellular environment is the principle that Fluorodeoxy *vitis vinifera* glucose positron emission tomography (Fluorodeoxy *vitis vinifera* glucose positron emission tomography, FDG‐PET) can be used to accurately diagnose tumors. Although the energy metabolism of the tumors is complex, reliable studies suggest that cancer cells are generally able to use the Warburg effect to preferentially acquire energy through aerobic glycolysis, while the intermediates of glycolysis are able to provide auxiliary pathways involved in the synthesis of various macromolecules, such as nucleotides, proteins, and lipids. These macromolecules are essential for cell growth and survival. MYC (MYC Proto‐Oncogene, BHLH Transcription Factor, MYC) is the major transcription factor in glucose metabolism, capable of upregulating the transcription [100] of almost all the metabolic enzymes in the glycolytic pathway, while the FOXOs transcription factor can inhibit MYC‐mediated glycolysis. The mechanism of this may involve the activation of the PI3K/AKT pathway, and abnormal activation of the PI3K/AKT signaling pathway is highly prevalent in malignant tumors. The PI3K/AKT signaling pathway mediates different metabolic processes, especially glycolysis, which can support cell growth and survival by upregulating glucose transporters and glycolytic enzymes. Abnormal activation of this signaling pathway promotes the expression of MYC, and simultaneous nuclear translocation of FOXOs with loss of transcriptional activity, and subsequently loses the antagonistic effect on MYC [101]. Moreover, the PI3K pathway can activate macropinocytosis, a nonselective form of massive endocytosis that can engulf exogenous macromolecules and deliver them to lysosomes for digestion, and this alternative nutrient acquisition strategy enables cancer cells to survive [102] in a nutrient‐poor microenvironment.

Amino acids are important for the proliferation, growth, and survival of tumor cells. Glutamine, an important one, is the second largest nutrient after glucose. In addition to participating in protein synthesis, glutamine also plays a variety of different anabolic roles in cancer cells. Studies have shown that cancer cells increase [103] uptake by upregulating plasma glutamine transporters ASCT 2/SLC1A5 (Alanine‐Serine‐Cysteine Transporter/Solute Carrier Family 1 Member 5, ASCT2/SLC1A5) and SN2/SLC38A5 (Nucleophilic Substitution/Solute Carrier Family 38 Member 5, SN2/SLC38A5), which are then converted to glutamate [104] in mitochondria by two forms of glutaminase (Kidney‐type glutamine amidase, GLS 1 and Liver‐type glutaminase, GLS 2). However, the MYC gene can positively control the expression of glycolytic enzymes, glutamine transporter protein, and glutaminase activity [100]. As mentioned previously, FOXOs have antagonistic effects on MYC [17, 105] and can inhibit glutamine absorption and utilization [101] by cancer cells. In addition, amino acids can also reverse regulate the activity of FOXOs [82]. What is more interesting is that GLS2 can inhibit the proliferation and migration of cancer cells. In hepatocellular carcinoma (HCC), GLS2 can inhibit in vitro proliferation and lung metastasis in xenograft mouse models and also reduce levels of reactive oxygen species (ROS) [104].

Dysregulation of lipid metabolism is highly prevalent in cancer, especially in HCC. The metabolism of lipids provides energy for cancer cells and is also an important component of cancer cells and a crucial signaling molecule in many cellular activities [106]. In normal physiology, the generation of fat is mainly limited to liver cells and adipocytes. FOXO1 is highly expressed in the liver and fat to maintain various liver functions and can also regulate lipid balance in the body in a bidirectional and benign manner [107]. It is well known that abnormal lipid metabolism is one of the very important risk factors for the occurrence and progression of HCC [108]. Reliable studies have shown that ATP‐citrate lyase (ATP‐citrate lyase, ACLY), acetyl‐CoA carboxylase (Acetyl‐CoA Carboxylase, ACC), fatty acid synthase (Fatty Acid Synthase, FASN), and stearyl‐CoA desaturase (Stearoyl‐CoA Desaturase, SCD) are considered key enzymes involved in lipid metabolism, which are commonly upregulated in HCC and associated with poor prognosis [109]. And these key enzymes are regulated by sterol regulatory element binding protein (sterol regulatory element binding proteins, SREBPs) transcription factors and then play the role of SREBPs transcription factors in regulating lipid metabolism [110]. SREBPs are a key transcription factor in the de novo synthetic fat (de novo lipogenesis, DNL) pathway in cancer cells. Other studies have shown that FOXO 1 is able to negatively regulate its [111, 112] expression by binding to SREBP2 and SREBP‐1c promoters to inhibit SREBP1 and SREBP2 gene transcription. Moreover, FoxO 1 also regulates the expression and activity [95] of two other major adipogenic transcription factors, PPARγ (Peroxisome proliferator‐activated receptor γ, PPARγ) and C/EBP α (CCAAT Enhancer Binding Protein α, C/EBPα).

### 3.3 Regulation of the Tumor Angiogenesis

As is well known, angiogenesis is one of the markers of solid tumors, and similarly, angiogenesis is crucial in the occurrence and development of HCC. Moreover, HCC is a typical highly vascularized solid tumor with an exceptionally rich vascular network and high angiogenesis characteristics, providing sufficient nutrition for the tumor while also endowing HCC with the characteristics of rapid progression, invasion, and metastasis. Transarterial chemoembolization (Transcatheter arterial chemoembolization, TACE) is a widely recognized and commonly used treatment for HCC worldwide, which indirectly demonstrates the importance of angiogenesis in the progression of HCC [113]. There is evidence to suggest that FOXOs are proangiogenic and antiangiogenic regulatory factors, playing a crucial role in angiogenesis. Firstly, FOXO1 knockout mice died during embryonic development due to vascular development defects, indicating that FOXO1 has a proangiogenic effect. On the other hand, FOXO3 knockout mice with hind limb ischemia showed an increase in capillary density 14 days after ischemia induction, indicating that FOXO3 is an important negative regulator of angiogenesis [114]. In addition, studies have shown that the deletion of any one or two genes of FOXO 1, FOXO 3, and FOXO 4 will lead to the emergence of hemangioma, and the simultaneous deletion of all three FOXOs can cause widespread vascular disease, even fatal, and is more pronounced in tissues such as the uterus and liver. This study effectively proves that FoxO1 is the most effective regulator of adult vascular homeostasis, while other FOXOs contribute less but are physiologically important. In addition, this study also investigated the significantly enhanced proliferation response of liver endothelial cells (Endothelial Cells, ECs) with three types of FOXOs deficiency to VEGF (Vascular endothelial growth factor, VEGF) and bFGF(Basic fibroblast growth factor, bFGF) (two effective proangiogenic cytokines) stimulation, while significantly reducing the sensitivity of endothelial cells (ECs) to the inhibitory cytokine TGF‐β1(Transforming growth factor‐β, TGF‐β1). Moreover, further research in this experiment suggests that liver endothelial cells (ECs) with three types of FOXOs deficiency were compared with normal ones, and it was found that 138 genes related to EC in the liver showed significant changes after FOXOs deficiency, with 89 upregulated and 49 downregulated. Sprouty2 (Sprouty RTK Signaling Antagonist 2, Sprouty2) and PBX1 (PBX Homeobox 1, PBX1) play important roles in EC growth and tube morphology and are the main effector molecules of FOXOs function in the endothelium [79]. Additional studies have shown that in HCC, the expression of FOXO1 is negatively correlated with intracellular levels of key participants in epithelial‐mesenchymal transition (EMT), which is a critical process in cancer metastasis [17, 115]. In addition, it was reported that increased FOXO 1 phosphorylation was positively correlated with higher microvascular area and higher expression of several angiogenesis‐related molecules, such as hypoxia‐inducing factor‐1 α (hypoxia inducible factor‐1 α, HIF‐1 α), VEGF, phosphorylated AKT (phosphorylated AKT), and nuclear factor κB (nuclear factor κB) [114, 116, 117]. More interestingly, the overexpression of FOXO 1 can inhibit MYC signaling, thus inhibiting the tumor energy metabolism function as well as the proliferation of endothelial cells [114]. It is clear that FOXOs have great potential for tumor angiogenesis in HCC.

### 3.4 Regulation of Tumor Immunity

The role of the immune system in tumors has two sides. On the one hand, it has a protective effect on the body; on the other hand, it has a certain promotional effect on tumors. While identifying and killing tumor cells, the complex immune environment of a tumor can establish a microenvironment conducive to tumor growth to promote tumor progression [118]. FOXOs play an important regulatory role in both physiological and pathological immune responses. Reliable studies have shown that FOXOs regulate immune‐related cell types, including keratinocytes, mucosal dermis, neutrophils, macrophages, dendritic cells, T cells, B cells, and natural killer cells (NK). In addition, FOXOs can regulate the expression of downstream target genes, including pro‐inflammatory signaling molecules, toll‐like receptors (TLRs), interleukin (Interleukin, IL)1, etc. β and tumor necrosis factor (TNF)‐α, chemokine receptors (C‐C chemokine receptor type 7, CCR7, and C‐X‐C Motif Chemokine Receptor 2, CXCR2), B cell regulatory factors (Aproliferation inducingligand, APRIL and B lymphocyte stimulator, BLYS), T cell regulatory factors (Foxp3 and CTLA‐4), and DNA repair enzymes (GADD45α). FOXOs act as cancer suppressors in tumors, and restoring the activity of FOXOs can modulate the cytotoxicity of CD8 + T and NK cells against tumor cells [119]. As is well known, during tumor development, newly released antigens are captured by dendritic cells (DCs) and presented to T cells, further activating the immune system, while FOXO1 typically exists in phosphorylated and inactive forms in DCs, ensuring their survival and proliferation [120]. Moreover, FOXOs are critical for T cell homeostasis, and conditional loss of FOXO 1 changes T cell homeostasis, causing a dramatic decline in T cell expansion while causing T cells that lack effector cytokines and exhibit an unresponsive signature [121, 122]. Recent studies have shown that FOXO 1 plays a key role in human T cell memory and that overexpressed FOXO 1 can increase the persistence of CART cell therapy and enhance the antitumor activity of memory reprogramming [123]. Moreover, it has been shown that FOXO 1 can not only target tumor cells, but also inhibit the progression of HCC by inducing and recruiting macrophages to antitumor differentiation, while inhibiting the expression of CD206, IL‐10, Arg‐1(Arginase 1, Arg‐1), and IL‐6 mRNA in macrophages [119]. On the other hand, the co‐expression and regulatory functions of FOXO1 and FOXO3 orchestrate different molecular mechanisms to ensure normal cell development and maturation [124]. The above studies indicate that FOXOs can exert antitumor immune function in HCC by regulating immune cells, which is another advantage of FOXOs.

## 4 Summary

**Figure 2:**
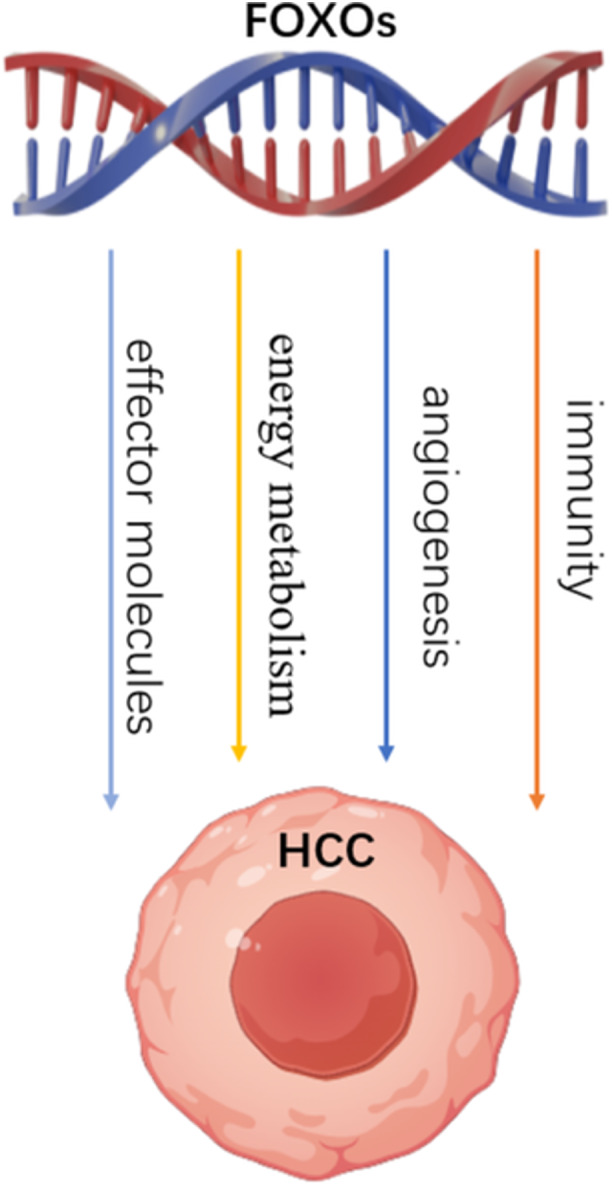
The Advantages of FOXOs in HCC.

According to the recent statistical results, the morbidity and mortality of HCC are increasing year by year, and other treatment methods besides surgery are difficult to benefit from in the long term [7]. In recent years, FOXOs have been further studied and found to play an important role in the occurrence and development of tumors. Therefore, the characteristics of FOXOs and their advantages in HCC (Figure 2) are integrated to provide a new idea for the mechanism research and antitumor treatment of HCC. FOXOs, as highly conserved transcription factors, are in a dysregulated state in various tumors. Their activation can control tumor cell proliferation by regulating the cell cycle, promoting tumor cell apoptosis, and coordinating various functions such as energy metabolism, angiogenesis, and immune regulation in HCC tumor cells to achieve antitumor goals. In addition, the activation of FOXOs can also enhance the efficacy of other treatments and improve the resistance of tumor therapeutic drugs in [123, 125], indicating that FOXOs play an active role in inhibiting cancer occurrence and treating tumors. In addition, studies have shown that FOXOs can inhibit HCC cell proliferation by inducing ferroptosis [126].

## 5 Outlook

Although substantial progress has been made in understanding the function and regulation of FOXO proteins, some researchers have clearly proposed that FOXOs can be used as new targeted therapies for HCC [127]. Substantial progress has been made in identifying compounds that activate or inhibit FOXO activity, and preliminary results have also been achieved in drug development, such as FOXO4‐DRI, carbenoxolone, and AS1842856. FOXO4‐DRI is a specific FOXO4‐p53 binding blocker that can selectively induce p53 nuclear rejection and endogenous apoptosis in senescent cells, thereby delaying organismal aging. Studies demonstrate that FOXO4‐DRI reduces the expression of senescence‐associated proteins, regulates apoptosis‐related proteins, consequently improving spermatogenic function and sperm quality in aged mice while exhibiting favorable safety profiles [128]. Furthermore, FOXO4‐DRI modulates the cell cycle of restrictively cultured keloid fibroblasts in vitro and selectively induces apoptosis in senescent keloid cells while altering FOXO4 cellular distribution [129]. FOXO4‐DRI also selectively eliminates senescent cells from in vitro expanded *Homo sapiens* chondrocytes [130]. Carbenoxolone, a derivative of glycyrrhetinic acid from Glycyrrhiza uralensis, has been shown to target FOXO family members (including FOXO1, FOXO3, FOXO4, and FOXO6) by binding to the winged‐helix DNA‐binding domain (DBD) of FOXO proteins. In addition, it suppresses FOXO3 transcriptional activity and counteracts FOXO3‐mediated chemoprotection in high‐risk neuroblastoma [131]. Glioblastoma multiforme (Glioblastoma multiforme, GBM) and basal‐like breast cancer (basal‐like breast cancer, BBC) exhibit FOXO1‐driven characteristics. Treatment with the FOXO1 inhibitor AS1842856 increases proapoptotic gene expression, enhances apoptosis, and reduces proliferation [132]. Nevertheless, there is still much that remains to be discovered; for example, FOXOs heavily rely on cell type and tissue environment to trigger different and even opposite functions, and it is important to determine the mechanisms by which FOXO factors specify gene expression and perform appropriate cellular functions accordingly. Therefore, a detailed understanding of the FOXO proteins and their biological properties in HCC is expected to provide new approaches for the treatment of HCC.

## Author Contributions

Gu Xiufeng designed this study and made a major contribution to the acquisition, analysis, and interpretation of data and drafting of the manuscript. Hu Zhiping was involved in interpretation and revision. Wang Jingzhi was involved in interpretation and revision. All authors read and approved the final manuscript. All authors have participated sufficiently in the work to take public responsibility for appropriate portions of the content and agreed to be accountable for all aspects of the work in ensuring that questions related to its accuracy or integrity.

## Conflicts of Interest

The authors declare no conflicts of interest.

## Data Availability

The data that support the findings of this study are available from the corresponding author upon reasonable request.
